# Adaptive designs: current status, future outlook

**DOI:** 10.1186/1745-6215-12-S1-A1

**Published:** 2011-12-13

**Authors:** Andy Grieve

**Affiliations:** 1 AptivSolutions , Robert-Perthel Strasse 77a, Cologne, Nordrhein-Westfalen, 50739, Germany

## 

The world of pharmaceutical statistics has taken adaptive designs to its heart - at least in theory. However despite the very large number of methodological publications and presentations at conferences there are still very few examples of adaptive designs in the medical literature. In this talk I will discuss why this might be the case and will suggest areas where these approaches are likely to be of greatest value in the future.

